# Effects of Exercise on the Inter-Session Accuracy of sEMG-Based Hand Gesture Recognition

**DOI:** 10.3390/bioengineering11080811

**Published:** 2024-08-09

**Authors:** Xiangyu Liu, Chenyun Dai, Jionghui Liu, Yangyang Yuan

**Affiliations:** 1 College of Publishing, University of Shanghai for Science and Technology, Shanghai 200093, China; liuxiangyu@usst.edu.cn; 2 School of Biomedical Engineering, Shanghai Jiao Tong University, Shanghai 200241, China; chenyundai@sjtu.edu.cn; 3 Institute of Science and Technology for Brain-Inspired Intelligence, Fudan University, Shanghai 200433, China; 4 School of Information Science and Technology, Fudan University, Shanghai 200433, China

**Keywords:** surface electromyography, hand gesture recognition, inter-session, machine learning

## Abstract

Surface electromyography (sEMG) is commonly used as an interface in human–machine interaction systems due to their high signal-to-noise ratio and easy acquisition. It can intuitively reflect motion intentions of users, thus is widely applied in gesture recognition systems. However, wearable sEMG-based gesture recognition systems are susceptible to changes in environmental noise, electrode placement, and physiological characteristics. This could result in significant performance degradation of the model in inter-session scenarios, bringing a poor experience to users. Currently, for noise from environmental changes and electrode shifting from wearing variety, numerous studies have proposed various data-augmentation methods and highly generalized networks to improve inter-session gesture recognition accuracy. However, few studies have considered the impact of individual physiological states. In this study, we assumed that user exercise could cause changes in muscle conditions, leading to variations in sEMG features and subsequently affecting the recognition accuracy of model. To verify our hypothesis, we collected sEMG data from 12 participants performing the same gesture tasks before and after exercise, and then used Linear Discriminant Analysis (LDA) for gesture classification. For the non-exercise group, the inter-session accuracy declined only by 2.86%, whereas that of the exercise group decreased by 13.53%. This finding proves that exercise is indeed a critical factor contributing to the decline in inter-session model performance.

## 1. Introduction

The application of electronic devices in modern society is increasingly widespread, thus facilitating the development of various human–machine interfaces (HMIs) [1]. HMI serves as the communication medium between human and machine, transmitting human control intentions to machines. Artificial intelligence algorithms [2] can analyze the information contained within HMIs to identify these intentions and then manipulate the machines to perform corresponding tasks, thus achieving human control over machines. Surface electromyography (sEMG) is a physiological signal generated by the muscles of the human body and collected on the surface of the skin [3]. It carries not only the physiological characteristic information of individuals [4,5], but also muscle activation information related to movement intentions [6]. This makes it a versatile human–machine interface. Since sEMG can intuitively capture muscle activities, it is widely applied in motion-intent recognition systems, such as prosthetic control [7,8] and gesture recognition [9,10]. Considering that gestures are a natural mode of human interaction, sEMG-based gesture recognition systems have significant application prospects.

As a gesture recognition system applied to real-world scenarios, good user experience is highly dependent on recognition accuracy. Previously, numerous researchers have devoted time to improving the model accuracy. To date, they have achieved excellent results with both traditional machine learning methods [11,12] and advanced deep learning algorithms [13,14]. However, when reused after a period of time, the system could perform poorly on the same task. Most current research attributes such inter-session degeneration mainly to the electrode shifts and environmental noise changes [15,16]. These factors lead to a variation in the overall distribution of sEMG data. Many existing studies have employed advanced machine learning algorithms to realize the recalibration or enhancement of sEMG data. Du et al. [17] established a domain adaption framework to reduce differences of the features between sessions, enhancing the accuracy from 62.7% to 82.3%. In Wang’s research [18], a triplet network was utilized to search for general features by deep-feature embedding, representing strong robustness in gesture recognition tasks across 15 sessions. From a more specific perspective, noise generated from changing environments and electrode shifts caused by different wearing styles are two of the most important factors reducing gesture recognition accuracy. In response to these two specific problems, researchers have designed various customized methods to enhance the generalization of the model across sessions. Jiang et al. [19] enhanced the noise resistance of the model by adding randomly generated channel perturbations to the sEMG feature maps in the training set, significantly improving the model performance in inter-session validation. For electrode shift, Zhang [20] quantified it by matching the overlapped regions of the muscular activation heatmaps between the training data and the testing data. After calibration according to the evaluation result, the hand-recognition accuracy in inter-session scenarios was dramatically increased by 25% on average.

Apart from external conditions, the characteristics of sEMG can also vary with the changes of individual physiological states, especially the states of muscles, since it is a physiological signal generated from muscles. During continued use, the muscle condition of the user can be influenced by various internal factors, such as growth, disease, fatigue, and exercise. Fatigue and exercise are the most common factors in daily life. For fatigue, it has been explored by many previous studies [21,22], and fatigue significantly reduces the accuracy of sEMG-based gesture recognition. However, its impacts are temporary and can be recovered with rest. In contrast, the changes caused by exercise are long term and can significantly interfere with system decision, yet are rarely investigated. Therefore, in this study, we chose exercise as the main factor for further exploration. We assumed that, for exercise groups, they would encounter continued performance deterioration of the model because of the changes in their sEMG features. This will contribute to terrible experiences for users. However, current research has not yet explored the impact of exercise on the performance of the gesture recognition system.

Therefore, in this study, we investigated the impact of muscle changes caused by exercise on the accuracy of an sEMG-based gesture recognition model. To evaluate this effect, we recruited six subjects as an exercise group to repeatedly perform the same gesture tasks, collecting their arm circumference and sEMG signals both before and after exercise. As a control group, another six subjects were recruited to perform the same tasks. Their arm circumference and sEMG signals were also collected during two sessions at the same interval as exercise group. Then, we used the Linear Discriminant Analysis (LDA) algorithm to establish a gesture recognition model for the ten most commonly-used gestures. Ultimately, when the model was trained with the pre-exercise dataset and tested on the post-exercise dataset, we found a significant decrease in the classification accuracy for post-exercise gestures. This result demonstrates that changes in muscle mass due to exercise are indeed a critical factor in the performance decline of sEMG-based gesture recognition models in inter-session scenarios.

## 2. Materials and Methods

### 2.1. Subjects

A total of 12 intact subjects, including 4 males and 8 females aged from 21 to 32 years old, were recruited in this experiment. All the subjects read the detailed experimental procedures and signed the informed consent form in advance. The experiment was approved by the ethics committee of Fudan University (approval number: BE2036).

### 2.2. Data Collection

**Figure 1:**
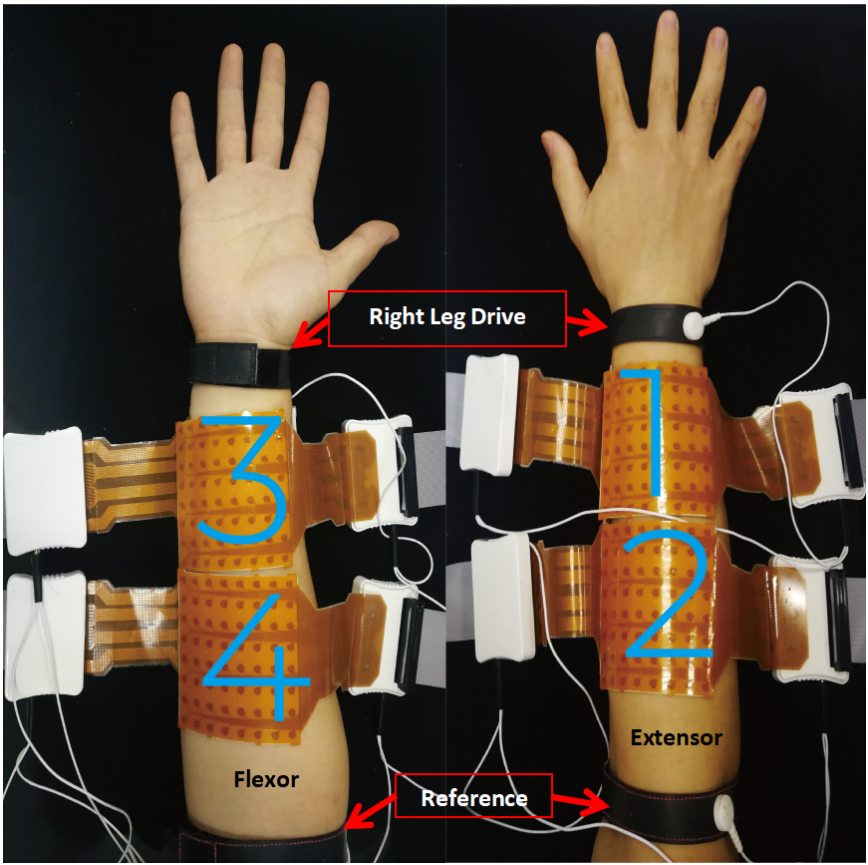
Electrode setup for the data collection. Numbers 1–4 denote the four 8×8 electrode arrays placed on the forearm.

Figure 1 shows the electrode and equipment configuration for the data collection of this experiment. Four 8×8 electrode arrays (256 channels in total) were used to record the high-density sEMG signals, with each of the 128-channel electrodes covering the extensor and flexor muscles of the forearm, respectively. To ensure the same electrode placement positions across sessions, we carefully marked the placement positions on the wrist of each participant with a permanent marker. Additionally, to prevent electrode displacement caused by the forearm movements (such as pronation and supination) during the experiment, we tightly secured the electrodes to the participants and fixed them with tape. Each electrode has an elliptical shape with a 5 mm major axis, 2.8 mm minor axis, and 10 mm inter-electrode distance. Two wet conductive bands were wrapped around the elbow and wrist as the reference electrode and right leg drive electrode, respectively. A commercial system Quattrocento (OT Bioelettronica in Torino, Italy) was used for signal acquisition with a sampling rate of 2048 Hz, an amplification gain of 150 and an A/D resolution of 16 bit.

**Figure 2:**
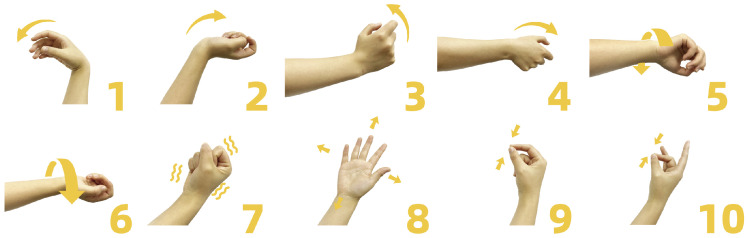
Ten gestures used in the experiment: (1) wrist flexion, (2) wrist extension, (3) wrist radial, (4) wrist ulnar, (5) wrist pronation, (6) wrist supination, (7) hand close, (8) hand open, (9) thumb and index finger pinch, and (10) thumb and middle finger pinch.

During the experiment, the subjects sat in a comfortable armchair with their arms naturally placed on the armrests. A computer monitor was positioned in front of the subjects, and a custom-built program guided them to perform 10 common gestures, as shown in Figure 2. The 10 gestures included (1) wrist flexion, (2) wrist extension, (3) wrist radial, (4) wrist ulnar, (5) wrist pronation, (6) wrist supination, (7) hand close, (8) hand open, (9) thumb and index finger pinch, and (10) thumb and middle finger pinch. For each gesture, subjects were required to repeat it six times at a comfortable speed for them (approximately 1 s). To avoid cumulative fatigue, a 2-s rest was provided between two repetitions, and a 5-s rest between two different gestures. Each subject performed the above experiment on two distinct days, approximately 7 days apart.

All 12 subjects were randomly and equally divided into two groups: the exercise group and the non-exercise group. The exercise group was required to undergo half an hour of standard upper limb strength training under the guidance of a professional coach every day. The training included wrist barbell flexion/extension/supination/pronation/radial deviation/ulnar deviation tasks, elbow barbell flexion/extension tasks, and hand grasp tasks. By contrast, the non-exercise group did not perform any strength training.

### 2.3. Data Preprocessing

#### 2.3.1. Filtering

Considering that the effective information frequency range of sEMG signals is between 10 and 500 Hz, we used a 10–500 Hz band-pass filter to process the raw data, retaining the useful information while filtering out motion artifacts and noise. Subsequently, we applied a series of notch filters at 50 Hz and its harmonics up to 400 Hz to eliminate power-line interference.

#### 2.3.2. Bad Channel Repairing

During data collection, bad channels with abnormal signal values can occur due to poor electrode contact or other issues. In this study, we defined a bad channel as one with signal values too large or small to be a normal sEMG signal or with a completely absent signal. More specifically, the signal values of the former fall beyond three standard deviations of the mean value of surrounding channels, and that of the latter are zero at any sampling point. To correct these bad channels, we replaced their signal values with the median of that from the surrounding channels.

#### 2.3.3. Feature Extraction

We extracted four typical sEMG features from the preprocessed signals with sliding windows, namely root mean square (RMS), wave length (WL), zero crossing (ZC), and slope sign change (SSC) [23]. These four features have been widely used in previous studies [23,24,25] and achieved good performance. In this study, we selected a window length of 0.5 s (1024 sampling points) and an overlap of 75%. Finally, five values of each feature were calculated for one channel of each sample.

### 2.4. Methods of Analysis

#### 2.4.1. Data Augmentation

For each individual feature, we first calculated an 8 × 8 feature map for each 8 × 8 electrode array and then upsampled it to 80 × 80 with bicubic interpolation. Subsequently, we randomly translated and rotated each 80 × 80 feature map. The horizontal and vertical translations were determined by random distances $d_{horizontal}$ and $d_{vertical}$, generated from a uniform distribution ranging from −15 to 15 mm. Positive values indicate rightward and upward translations of the electrode array in Figure 1, while negative values indicate the opposite directions. Then, each feature map was rotated by a random angle α, where α was uniformly distributed between −15° and 15°, with the sign indicating the direction of rotation (positive for clockwise; negative for counterclockwise). Finally, we downsampled the shifted 80 × 80 feature maps back to 8 × 8. For each original sample, we applied data augmentation to generate two augmented samples, finally generating a training set three times larger than the original. Feature map shifting was performed independently for each 8 × 8 electrode array, each individual feature, and each training sample. This data-augmentation method refers to [16], increasing the diversity of the training data and simulating variations due to electrode shifts.

#### 2.4.2. Linear Discriminant Analysis

To compare the gesture recognition accuracies of the model in different sessions, we selected Linear Discriminant Analysis (LDA) [26], a typical machine learning algorithm, as the classifier in this study.

### 2.5. Validation Protocols

#### 2.5.1. Intra-Session Validation

In intra-session validation, for each subject, 80% of the samples of each gesture from the same session (pre-exercise or post-exercise) were randomly selected as the training set, and the remaining 20% were used as the test set. To minimize the impact of randomness on the final results, 10 repeated experiments were conducted for each subject. The final accuracy listed in the results is the average of 10 repeated experiments across all subjects in the same group.

#### 2.5.2. Inter-Session Validation

In inter-session validation, for each subject, all samples from the first session (pre-exercise) and the second session (post-exercise) were used as the training set and the test set, respectively. The final accuracy listed in the results is the average of all subjects in the same group.

### 2.6. Statistical Analysis

After conducting the Shapiro–Wilk test, the results indicated a deviation from a Gaussian distribution. Consequently, non-parametric tests were employed for statistical analysis. Specifically, the Wilcoxon signed-rank test was selected as the statistical method for comparing the two groups in this study.

## 3. Results

### 3.1. Two-Dimensional Heat Map of Muscle Activation

**Figure 3:**
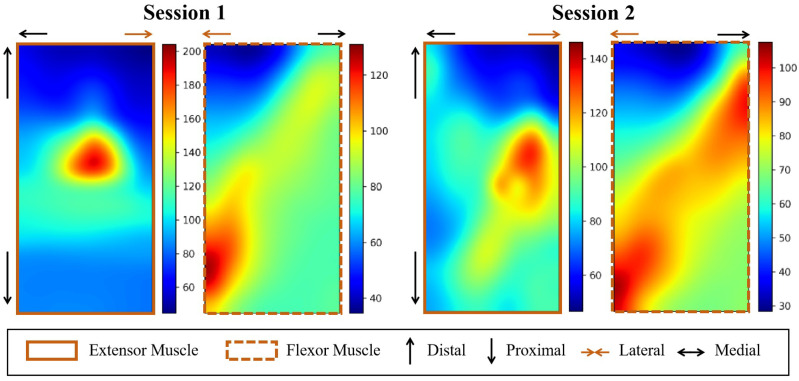
Two-dimensional heat map of muscle activation before and after exercise. Each map presents the RMS of sEMG signals. Brighter pixels denote more active muscle groups. For better visualization, the original 16×16 maps are upsampled to 100×100 via bicubic interpolation.

Figure 3 shows the activation heat maps of flexor and extensor muscles for a representative subject before (Session 1) and after (Session 2) exercise when performing the thumb and middle finger pinch task. We can observe that the muscle activation area becomes larger after exercise due to the strengthening of the muscle fibers. However, the relative position and active pattern remain similar.

### 3.2. Intra-Session Recognition Accuracy of Exercise and Non-Exercise Groups

**Figure 4:**
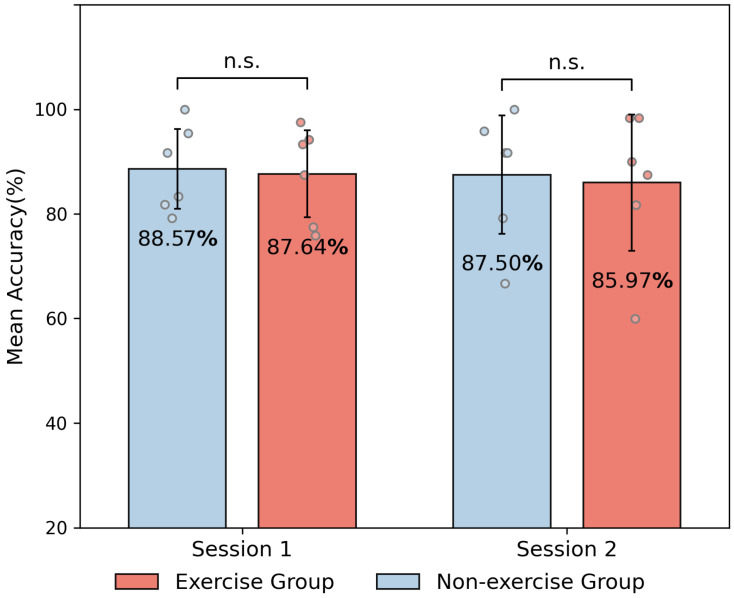
Mean classification accuracies (%) within sessions for two groups. Statistical tests were conducted between the two groups for each session. ‘n.s.’ denotes that no statistical significance was found between exercise and non-exercise group.

Figure 4 shows that the classification accuracies for both the exercise and non-exercise groups in Sessions 1 and 2 are around 85% to 88% during the intra-session classification. There was no significant difference in the intra-session accuracy between the two groups in both session. This indicates a high consistency in feature representation within the two sessions for both groups.

### 3.3. Inter-Session Recognition Accuracy of Exercise and Non-Exercise Groups

**Figure 5:**
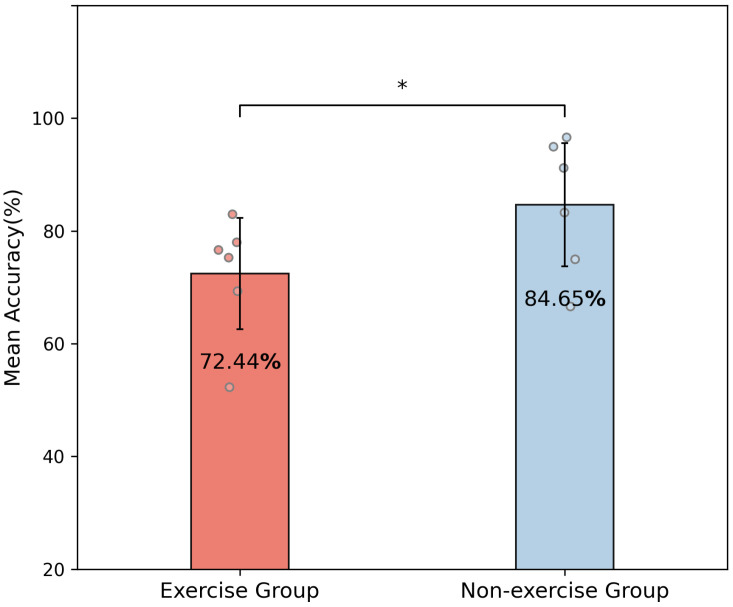
Mean classification accuracies (%) in the inter-session scenario for two groups. A statistical test was conducted between the two groups. ‘*’ denotes a significant difference between the two groups, with a *p*-value between 0.01 and 0.05.

Figure 5 shows that the mean inter-session classification accuracy for the non-exercise group was significantly lower than that for the exercise group (0.01 < *p* < 0.05). The difference in mean classification accuracy between the two groups was 12.21%. This indicates that the feature representation of the data between the two sessions was more similar for the non-exercise group. In contrast, the lower inter-session accuracy for the exercise group suggests a greater change in feature representation across different sessions. Given that the experimental paradigm and environment were kept constant, the significant difference in accuracy between the two groups is most likely attributed to the exercise factor.

**Figure 6:**
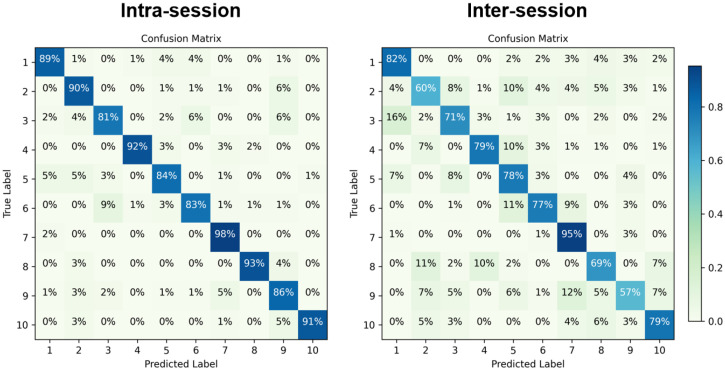
Confusion matrix for intra-session and inter-session gesture recognition. The numbers 1 to 10 represent the index of hand gestures shown in Figure 2. Note that the value in row i and column j represents the probability that gesture i (i = 1, 2, …, 10) is recognized as gesture j (j = 1, 2, …, 10).

**Figure 7:**
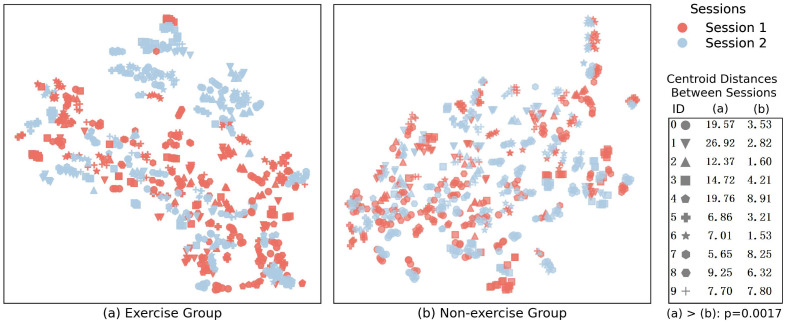
The visualized data distribution of exercise group and non-exercise group. The bottom-right corner of the figure exhibits the centroid distances of the ten categories between two sessions for the exercise and non-exercise groups. A larger distance indicates a more significant deviation in data distribution between sessions. A *p*-value of less than 0.05 indicates that the data distribution deviation in the exercise group is significantly greater than that in the non-exercise group.

Figure 6 shows the confusion matrix for the intra-session and inter-session gesture recognition. Compared to the intra-session, there are different degrees of decline in the recognition accuracy of all the gesture for inter-session conditions. Specifically, wrist extension, hand open, and thumb and index finger pinch show the most significant decline. These gestures are mostly related to extensor muscles, indicating that exercise may have a more pronounced effect on the extensor muscle group. Additionally, Figure 7 shows the data distribution of each gesture for both the exercise and non-exercise groups for the different sessions. We can clearly observe a deviation in the data distribution between the two sessions for the exercise group, leading to a decline in recognition accuracy.

### 3.4. Trends in Recognition Accuracy and Biceps Circumference

**Figure 8:**
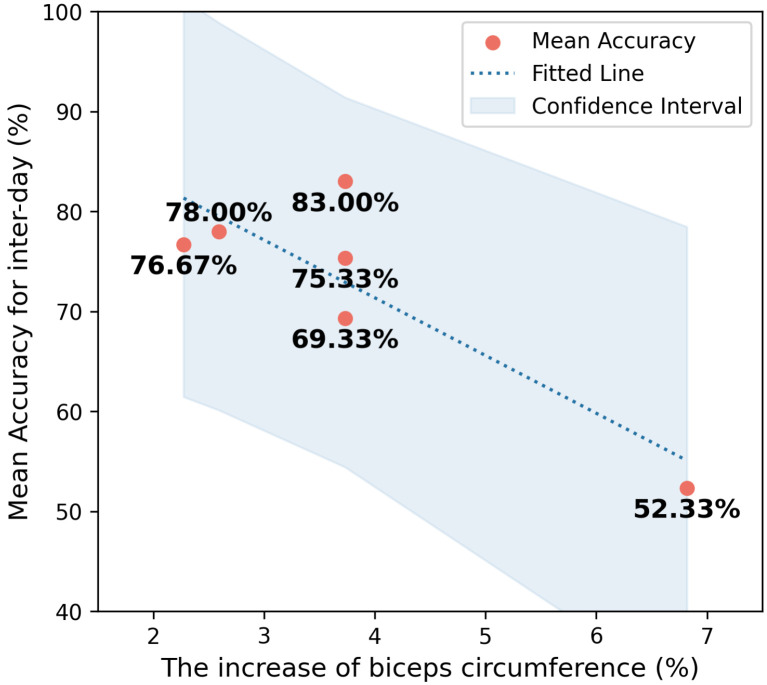
Relationship between the increase in biceps circumference (%) and mean inter-session classification accuracy (%). Each red dot represents the mean accuracy for a specific increase in biceps circumference. The dotted line represents the fitted trend, and the shaded area indicates the confidence interval.

In this study, we evaluated the changes in muscle state by biceps circumference. Figure 8 shows a negative correlation trend between the increase in biceps circumference and mean inter-session accuracy. An ordinary linear regression was performed on the increase in biceps circumference and mean inter-session accuracy. Additionally, a statistical hypothesis test was conducted on the trend term. The results indicate that the trend of decreasing mean inter-session accuracy with increasing biceps circumference is significant (*p* = 0.028).

## 4. Discussion

sEMG-based gesture recognition is a commonly used human–machine interaction interface. Users can control external terminals (such as robotic arms, prostheses, exoskeletons, etc.) using sEMG signals from different gesture commands. Such a human–machine system requires training a model that maps each gesture label to its corresponding EMG signals. Previous studies [27,28] have found that high recognition accuracy can only be achieved when users calibrate the system before each use. However, model calibration typically involves many tedious operations such as additional data collection or model retraining, greatly reducing users’ convenience. Therefore, we hope that users do not need to calibrate the system again when using it in different sessions once the initial calibration completed.

The user experience of a sEMG-based human–machine system mainly depends on accurately recognizing sEMG patterns generated by different gestures. The high-precision recognition outcomes require the consistency of the sEMG patterns generated by the same gesture for model training and testing. The data for model training and testing under the inter-session scenarios are typically acquired at different timings. Previous studies [15,19] have explored the effect of the system on gesture recognition accuracy when users use it for multiple sessions. In such cases, many external and internal factors can cause changes in the sEMG patterns, such as electrode sensor shifts, variations in background noise, and muscle fatigue. These common factors have been widely discussed in previous studies and can affect recognition accuracy to some extent. Although different studies may have different experimental protocols, the impact of electrode shift depends on the degree of the shift, and usually affects the accuracy decline by 3–15% [29]. Background noise and fatigue have a smaller impact, usually between 3 and 5% [15,19]. Moreover, previous studies and our research have found that if these common factors are minimized, the decrease in inter-session accuracy is minimal. However, muscle exercise is another factor that is a very common factor in daily life, with rarely explored in previous studies. We found that muscle exercise may have a significantly greater impact on recognition accuracy decline than other common factors, as indicated by an approximately 13.5% decrease in our study. The potential reason may be that exercise changes the structure of the muscles and alters the way users exert force, leading to significant changes in sEMG patterns when performing the same gestures. Future work could focus on minimizing the impact of exercise through sEMG feature selection, data preprocessing optimization, and advanced classification modeling [16].

Accordingly, this study investigated the impact of exercise on the accuracy of gesture recognition for intra-session and inter-session conditions. For the intra-session conditions, exercise does not affect the recognition accuracy. This may be because, although exercise changes muscle fibers, the muscle state remains stable within the same session. In other words, as long as the EMG data used for model training and data for gesture recognition testing are collected under the same muscle state, recognition accuracy will not decline, and the strength of the muscles does not affect recognition accuracy. For the inter-session conditions, the control group (non-exercise group) experienced only about a 2% decline in recognition accuracy. As this experiment strictly ensured the consistency of electrode placement on multiple sessions, the slight decline in the non-exercise group may be due to environmental noise and other background inference, proving the robustness of the EMG human–computer interaction system for multiple-session use. However, exercise significantly impacts recognition accuracy, with the accuracy of exercise group dropping by more than 15%. Exercise undoubtedly affects the state of muscle fibers, which participates in the generation of sEMG, ultimately leading to a decline in accuracy for the inter-session condition. Additionally, through regression analysis, we find that, if the muscles (muscle circumference) become stronger, a greater decline in accuracy is achieved, with a trend of an approximately 5% drop in recognition accuracy for every 2% increase in muscle circumference.

In summary, muscle changes caused by exercise affect the accuracy of EMG human–computer systems in practical use, and the degree of accuracy decline cannot be ignored. Future studies may address this issue from the following perspectives:

1. Exploring the sEMG features insensitive to muscle fiber changes, such as motoneuron discharge information or frequency domain information.
2. Utilizing advanced machine learning algorithms. Recent algorithms, such as transfer learning may provide solutions. This algorithm can train a model based on non-exercise data and then fine-tune it with a small amount of exercise data to improve recognition accuracy.

Unfortunately, due to the extended duration of the entire experimental period, we have currently collected data from only 12 participants, resulting in a relatively small dataset for this experiment. In future research, we hope to validate our hypotheses with more data to obtain more general results. Additionally, in this study, we only proved that exercise leads to a decline in the inter-session performance of the model. However, we have not yet proposed an effective solution to this problem. In our subsequent research, we hope to employ more advanced data processing methods and deep learning algorithms to improve the model performance in scenarios where users engage in continuous exercise.

## Data Availability

The original contributions presented in the study are included in the article, further inquiries can be directed to the corresponding author/s.
